# Tracing Early Neurodevelopment in Schizophrenia with Induced Pluripotent Stem Cells

**DOI:** 10.3390/cells7090140

**Published:** 2018-09-17

**Authors:** Ruhel Ahmad, Vincenza Sportelli, Michael Ziller, Dietmar Spengler, Anke Hoffmann

**Affiliations:** Max Planck Institute of Psychiatry, Translational Psychiatry, 80804 Munich, Germany; ruhel_ahmad@psych.mpg.de (R.A.); vincenza_sportelli@psych.mpg.de (V.S.); michael_ziller@psych.mpg.de (M.Z.); hoffmann@psych.mpg.de (A.H.)

**Keywords:** patient-specific iPSCs, schizophrenia, early neurodevelopment, differentiation, neurotransmission, mitochondria, micro-RNA, neuroleptics

## Abstract

Schizophrenia (SCZ) is a devastating mental disorder that is characterized by distortions in thinking, perception, emotion, language, sense of self, and behavior. Epidemiological evidence suggests that subtle perturbations in early neurodevelopment increase later susceptibility for disease, which typically manifests in adolescence to early adulthood. Early perturbations are thought to be significantly mediated through incompletely understood genetic risk factors. The advent of induced pluripotent stem cell (iPSC) technology allows for the in vitro analysis of disease-relevant neuronal cell types from the early stages of human brain development. Since iPSCs capture each donor’s genotype, comparison between neuronal cells derived from healthy and diseased individuals can provide important insights into the molecular and cellular basis of SCZ. In this review, we discuss results from an increasing number of iPSC-based SCZ/control studies that highlight alterations in neuronal differentiation, maturation, and neurotransmission in addition to perturbed mitochondrial function and micro-RNA expression. In light of this remarkable progress, we consider also ongoing challenges from the field of iPSC-based disease modeling that call for further improvements on the generation and design of patient-specific iPSC studies to ultimately progress from basic studies on SCZ to tailored treatments.

## 1. Introduction

Schizophrenia (SCZ) has a lifetime prevalence of ≈1% worldwide [1] and confers substantial mortality and morbidity. Life expectancy of patients with SCZ is reduced by 15 to 30 years with no cure available yet [2,3]. In 2017, ≈21 million were diagnosed with SCZ [4]; for comparison, ≈35 million were diagnosed with cancer. A century of investigation on the biology underlying SCZ has provided limited insight with multifarious, partly contradictory, results, but no conclusive disease mechanism has been established. However, epidemiological and clinical evidence supports a neurodevelopmental component in SCZ [5] that may increase later susceptibility for disease. Accruing evidence [6] on the genetic overlap between SCZ and neurodevelopmental disorders provides further support for this hypothesis.

Neural cells that are generated from patient-specific induced pluripotent stem cell (iPSCs) capture a donor’s genotype, including disease related genetic risk factors, known and unknown. Analysis of their effects on cellular and molecular endophenotypes during early neurodevelopmental stages in vitro is therefore postulated to cast new light on the potential neurodevelopmental origin of SCZ. 

To support this hypothesis, we will consider current evidence for an early neurodevelopmental component of idiopathic SCZ by summarizing clinical course, epidemiological, neuroimaging, and histological data, and recent insight into SCZ’s complex genetic architecture. Thereafter, we will discuss how neuronal cells from early developmental stages differ between patients with SCZ and healthy donors, how they respond to neuronal activation and clinically used drugs, and whether molecular and cellular alterations in patient-derived cells can bridge SCZ to early brain development. Lastly, we consider ongoing challenges to patient-specific disease modeling and critical steps that are required to advance the field further.

The literature selection process for this review was performed in the databank PubMed using combinations of the search terms “schizophreni*”, “induced pluripotent stem cell*”, “genetic*”, and “psychosis” with date limits from 2007 (first report on iPSCs [7]) to August 2018. Additional searches included scrutiny of similar articles suggested by PubMed, of references from the identified publications, and of citatory publications identified by Google Scholar^®^.

### 1.1. The Neurodevelopmental Hypothesis of SCZ

In the early 20th century, the psychiatrist Manfred Bleuler [8] coined the term SCZ for a profound disorder that is characterized by disturbances of association, affect, ambivalence, and autistic isolation. The World Health Organization’s International Pilot Study of SCZ [9] weighted psychotic symptoms more strongly than Bleuler since they can be more easily distinguished from the normal range of experiences: Psychotic or positive symptoms [10,11] refer to common hallucinations (hearing, seeing, tasting, smelling, or feeling things that are not there) and delusions (fixed false beliefs or suspicions that are firmly held even when there is evidence to the contrary). Positive symptoms include also disorganized thinking (difficulties in keeping track of thoughts and conversations, drifting from one idea to another, poor concentration, jumbled, or confused thoughts). Negative symptoms of SCZ [10,11] appear several years before individuals experience a first acute schizophrenic episode and are often referred to as the prodromal period of SCZ: Patients lack interest and motivation in life, withdraw socially, and do not care about their personal appearance. These symptoms evolve gradually and slowly get worse.

The serendipitous discovery of neuroleptics in the second half of the 20th century transformed the treatment of SCZ and revived interest in its neurochemical basis. The psychosis-inducing effects of dopamine releasing drugs (e.g., amphetamine) and the anti-psychotic efficacy of dopamine D2-receptor blockers, led to the hypothesis of SCZ as a dopamine disorder [12]. Early neuroleptics (e.g., chlorpromazine and haloperidol) were replaced with time by ‘atypical’ neuroleptics owing fewer extrapyramidal side effects (e.g., tremor and rigidity). Although both groups reduce consistently acute hallucinations and delusions, relapse rates are still as high as 80% [13], and less than 14% of the patients experience sustained recovery in the first five years following a psychotic episode. A plausible explanation is that negative symptoms, especially cognitive deficits, are largely unresponsive to neuroleptics. These deficits may reflect altered glutamatergic signaling as evidenced by the psychomimetic effects [14] of the glutamatergic *N*-methyl-d-aspartate (NMDA) antagonists phencyclidine and ketamine in healthy subjects. In any case, improved treatments for negative symptoms are urgently needed to prevent frequent relapse, poor recovery, and insidious hospitalization.

In 1957, Barbara Fish [15,16] hypothesized that faulty timing and integration of development may contribute to rare forms of early onset SCZ. About 30 years later, Lewis, Murray, and Weinberger [17,18,19] extended this hypothesis to idiopathic SCZ: Perturbations in normal brain development increase the vulnerability towards cellular and molecular changes that are associated with development and/or early experiences and manifest time-delayed as SCZ in early adulthood. Importantly, early alterations remain largely latent until critical processes of normal maturation call into operation the damaged structures. 

In support of this hypothesis, various prenatal or perinatal risk factors, comprising infection in the 1st and 2nd trimester [20], cytokine exposure [21], perinatal injury [22], and maternal malnutrition during famine [23,24], confer a modest (<2-fold), and non-specific increase in risk for SCZ. As of yet, the causal relationship between these early risk factors and adult neuropathology remains unknown given secondary effects from disease processes, therapy, or life history. 

Longitudinal population- and family-based studies [25,26,27,28,29,30,31] have associated delayed development, neurocognitive and neurobehavioral deficits, and poor intelligence with an increased risk for SCZ indicating that SCZ evolves from a vulnerable brain. However, the underlying cause(s) of these findings as well as their progression over the course of the disease remain uncertain [32,33,34]. 

Meta-analysis of neuroimaging studies on patients with SCZ confirmed the enlargement of lateral and third ventricles, and smaller cortical and gray matter volumes. These alterations concur with relatively smaller volumes of the medial temporal lobe and the thalamus [35,36,37,38]. Macroscopic differences in neural structures are readily apparent in discordant monozygotic twins [39], in first-episode untreated patients, in high-risk and unmedicated individuals [40], and are not seen in bipolar patients to the same extent [41]. Since most of these studies refer to adult case/control cohorts, future neuroimaging needs to be placed in the context of normative developmental and atrophic changes in neural structures relevant to SCZ to inform on the role of early neurodevelopment. At the microscopic scale, contemporary histological studies have established cytoarchitectural anomalies, such as smaller cortical and hippocampal pyramidal neurons, decreases in cortical and hippocampal synaptic markers, and decreased dendritic spine density [42,43].

### 1.2. The Genetic Architecture of SCZ

Heritability for SCZ is high with estimates about 60% in two national family studies [44,45] and about 80% in twin studies [46,47]. Genome-wide association studies (GWAS) [48] have detected many common genetic variants of small effect size that explain between one-third and one-half of the genetic variance in liability. GWAS are based on DNA chips that contain millions of single nucleotide polymorphisms (SNPs) for high genome coverage. A recent landmark meta-analysis [49] of common SNP variants from 36,989 patients with SCZ and 113,075 controls identified 128 common variant associations covering 108 independent loci that fulfilled genome-wide statistical significance (e.g., 5 × 10^−8^). These risk loci spanned multiple regions enriched in genes that are relevant to glutamatergic neurotransmission, neuronal calcium and G-protein coupled receptor signaling, neuronal ion channels, synaptic function and plasticity, and several neurodevelopmental regulators. Genes residing in these risk loci are expressed in pyramidal excitatory neurons and a subset of GABAergic (γ-aminobutyric acid) interneurons [50,51], but substantially less in progenitor or glial cells. Of note, 93 of these risk loci have been replicated in a recent GWAS study [52] that identified additionally 52 new loci and highlighted six independent gene sets that are associated with SCZ: targets of FMRP (fragile X-mental retardation protein), abnormal behavior (in mice), 5-HT_2c_ receptor complex, abnormal nervous system electrophysiology, voltage-gated calcium channel complexes, and long-term potentiation (LTP). Moreover, a recent genome-wide association study [53] for shared risk across major psychiatric disorders (including SCZ) has implicated fetal neurodevelopment as a key mediator of vulnerability. The researchers identified four genome-wide significant loci covering variants predicted to regulate genes expressed in radial glia cells and interneurons in the developing cortex during midgestation.

Risk-associated SNPs typically mapped to non-coding genomic regions equally represented by intergenic and intronic regions [54] and are not necessarily the causal genetic variant underlying the association nor do they identify the causative gene(s). Future studies still need to identify those SNPs that encode a regulatory function and contribute causally to SCZ [55]. 

Beyond common variants, rare genomic variants associate as well with SCZ. Whole exome sequencing studies have detected rare coding mutations in voltage-gated calcium channels, NMDA receptor, and ARC-associated scaffold with no single causative gene as of yet identified [48,56,57]. Likewise, combined meta-analysis (21,094 patients with SCZ and 20,227 controls) detected in a small fraction (1.4%) of the cases genome-wide significant association with copy number variants (CNVs). The aggregate CNV burden was enriched for candidate genes encoding synaptic function (OR = 1.68, P = 2.8 × 10^−11^) and neurobehavior in mice. 

As of yet, risk SNPs and CNVs identified so far explain only a small fraction (<5%) of SCZ heritability [58] and many are thought to exist among common variants with effect sizes far below genome-wide statistical significance [59]. Accordingly, the recently suggested “omnigenic” model hypothesizes that gene regulatory networks are sufficiently interconnected such that all genes expressed in disease-relevant cells contribute to the functions of core disease-related genes [60]. 

Conclusively, future studies are urgently needed to link risk variants-to genes-to function in order to understand the biology underlying SCZ. In this respect, iPSC-based studies can provide an important tool to study the regulatory effects of SNP variants, candidate genes, and the overall effect of candidate genes and interconnected networks, known and unknown, on cellular and molecular endophenotypes in disease relevant human cells. 

### 1.3. Early Brain Development

**Figure 1:**
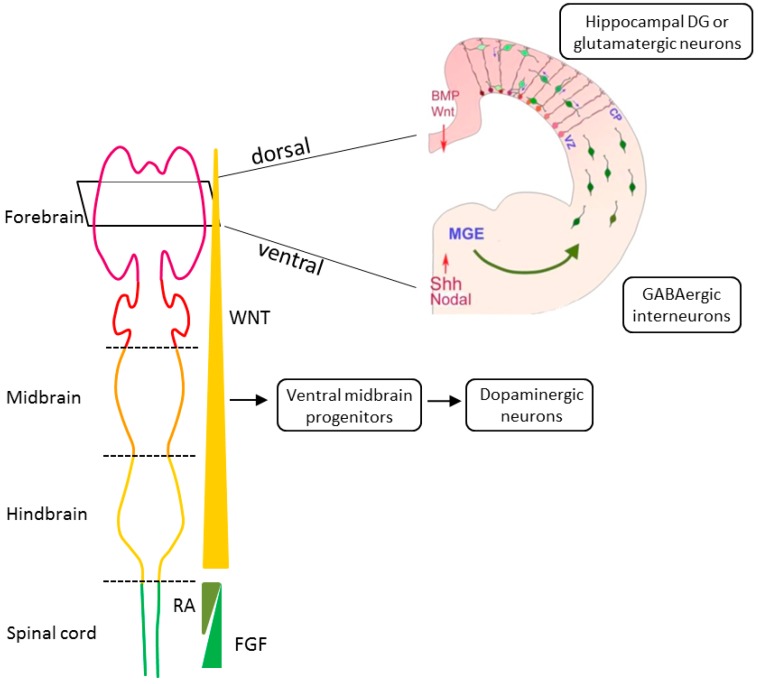
Tracing early neurodevelopment. Key morphogens in anterior–posterior patterning are WNT (wingless), FGF (fibroblast growth factors), and RA (retinoid acid). Gradients of these morphogens regulate differentiation into specific types of neural progenitor cells of forebrain (purple), midbrain (orange), hindbrain (yellow), and anterior spinal cord (green). Key morphogens in dorsal–ventral patterning are gradients of BMP (bone morphogenetic proteins), WNT, SHH (sonic hedgehog), and Nodal. BMP and WNT determine the dorsal fates of neural progenitor cells, whereas SHH and Nodal determines ventral fates. Different types of neural cells for tracing early neurodevelopment in schizophrenia (SCZ) are shown: Ventral midbrain progenitors give rise to tyrosine hydroxylase-positive dopaminergic neurons. Neural progenitor cells (green) from the ventricular zone (VZ) of the dorsal telencephalon generate all excitatory glutamatergic pyramidal neurons. Dorsal progenitors use radial glia cells (red) as scaffold to migrate radially to the cortical plate (CP) and from there to destined cortical layers. Interneurons (green) are derived from the ventral neurogenic zone termed medial ganglion eminence (MGE) and migrate tangentially to the pallium. There, interneurons can use radial glia cells to ascend to the cortical plate as is the case for inhibitory GABA-ergic interneurons or to descend to the ventricular zone. Differential interactions between subsets of interneurons and the radial glial scaffold are indicated by shades of green and red, respectively. On top of this, local guidance cues that are indicated by a gradient of pink can influence interneuron positioning as shown by blue arrows. Part of Figure 1 is reprinted from K. Sue O’Shea and Melvin G. McInnis, Neurodevelopmental origins of bipolar disorder: iPSC models; published by Molecular and Cellular Neuroscience, volume 73, pp. 63–83, 2016, with permission from Elsevier.

In light of accruing evidence for a neurodevelopmental origin of SCZ, we consider the next major principles of early brain development. The outer embryonic layer gives rise to the neuroepithelial cells of the neural plate that invaginates to form the neural tube. Upon closure, the most rostral region or the neural tube expands to produce the telencephalic vesicles whose lumen will become the lateral ventricle of the cerebral hemispheres, while the enclosing cell layer develops into the cerebral cortex, basal ganglia, and hippocampus [61] (Figure 1). Initially, the telencephalon consists of the dorsal (pallium) and ventral (subpallium) neurogenic zones that are of particular interest to SCZ: Neural progenitor cells (NPCs) from the dorsal zone produce all of the cortical excitatory pyramidal neurons that migrate radially to reach destined cortical layers. By contrast, NPCs from the ventral zone produce most of the cortical inhibitory neurons that migrate tangentially to reach their final cortical position [62] (Figure 1).

NPCs firstly undergo proliferative, symmetric divisions that increase the NPC population and drive expansion of the human neocortex [63]. Subsequently, the onset of asymmetric divisions results in increasing number of committed NPCs with only a small fraction of neural stem cells (NSCs) persisting into adult life in the ventral subventricular zone.

**Figure 2:**
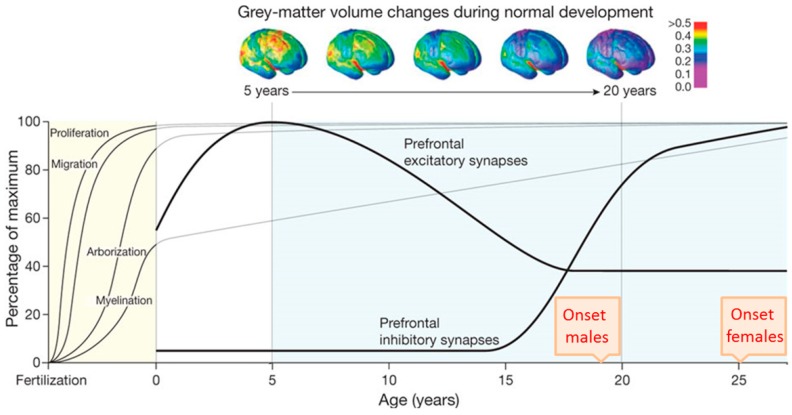
Temporal course of selected neurodevelopmental processes. The *x* axis depicts time from fertilization to young adulthood and the *y* axis shows the relative percentage of the maximum. The average age of schizophrenia (SCZ) onset in males and females in late adolescence and early adulthood, respectively, is indicated. Neurogenesis and subsequent migration of neurons to the cortex begin within a few weeks of gestation in humans and is completed around birth. Dendritic arborization and myelination continue postnatally towards adolescence and beyond. The progressive reduction of grey-mater volume detected with longitudinal neuroimaging (top) is thought to result from the combined effect of pruning of the neural arbor and myelin deposition. The formation of prefrontal excitatory synapses reaches a maximum in childhood at five years and declines thereafter until adolescence. By contrast, the formation of prefrontal inhibitory synapses strongly increases from 15 years onward through adolescence and levels off in early adulthood. Deregulation of the cortical excitatory-inhibitory balance is hypothesized to contribute to the development and manifestation of SCZ. Adapted from Thomas R. Insel, Rethinking Schizophrenia, published by Nature, 2010.

Human brain development begins in the third gestational week with the differentiation of NPCs and continues through at least late adolescence (Figure 2). Neural tube formation, neural patterning, and NPC differentiation occur in embryonic and early fetal periods, followed by neuron production, migration, and differentiation in later fetal and early postnatal periods. Postnatally, regressive and progressive neuronal processes, remodeling of synaptic contacts and circuitries, and myelination take place [61,62,64]. By early adulthood, cortical circuits are refined through the pruning of excitatory synapses, proliferation of inhibitory circuits, and remodeling of pyramidal dendrites [65,66]. These processes fine-tune the excitatory-inhibitory cortical balance and take place during the time period in which early and adolescent deficits in neurodevelopment are played out and manifest SCZ.

### 1.4. The Rational of iPSC-Based Disease Modeling

Today, human iPSCs are routinely established from skin biopsies or peripheral blood mononuclear cells [67,68]. Human iPSCs retain the unique genetic signature of the donor and they provide insight into the relationship between the donor’s genotype and an in vitro endophenotype. For case/control studies, iPSCs can be differentiated into virtually any cell [69], including disease relevant neurons and astroglia in order to re-enact altered trajectories of brain development in a diseased individual. Unlike postmortem brain tissue, human iPSCs are not confounded by secondary disease processes, therapy, or life history, and they offer a unique opportunity to study genetic programs that operate in the prenatal and postnatal brain. 

Importantly, comprehensive RNA expression profiling [70,71,72,73,74] of human brain tissues from early embryonic to late adult postmortem stages has shown that neuronal cells produced from iPSCs closely recapitulate the progression from early embryogenesis to late fetal periods in vitro and yield neuronal cells of various stages of maturity. In light of these findings, caution should be taken to extrapolate from current iPSC-studies to findings in adolescents and adults with SCZ. On the other hand, immature neurons and networks express molecules and processes that are not operative in the adult and they follow a crucial developmental sequence that is instrumental in the formation of functional entities. Neuronal cells that are derived from case/control iPSCs provide an extraordinary opportunity to access early developmental perturbations in combination with functional studies. These benefits make iPSCs a versatile tool to explore early molecular and cellular endophenotypes from patients with SCZ in disease-relevant cell types and to assess cause-effect relationships.

### 1.5. Tracing Early Neurodevelopment in SCZ

The first report on iPSC-derived neuronal cells from patients with SCZ appeared as early as 2011 [75]. Since then the number of iPSC-based studies has increased steadily. For clarity, we discuss these studies with a focus on common themes (neurodevelopment and differentiation, oxidative stress, and microRNAs), rather than by their chronological order and summarize experimental approaches and key findings in a tabular format. 

#### 1.5.1. Neurodevelopment and Differentiation of SCZ iPSC-Forebrain Neurons

In 2011, Brennand and coworkers [75] firstly reported an iPSC-based case/control study on familial SCZ. Primary fibroblasts were reprogrammed by inducible lentivirus and quality controlled (Table 1). One patient manifested early-onset SCZ, while the remaining three patients were from two families in which all offspring and one parent were affected by a disease from the schizophrenic spectrum (i.e., SCZ, schizoaffective disorder or schizoid personality disorder) (Table 2).

SCZ iPSCs were differentiated with similar efficiency relative to age- and ancestry-matched healthy donors into NPCs and were subsequently into mixed forebrain neurons (>60% glutamatergic, ≈30% GABAergic, <10% dopaminergic) that were co-cultured with human cerebellar astrocytes to further maturation (Table 2 and Table 3).

Neuronal connectivity of iPSC-derived case/control neurons was assessed by a modified rabies virus whose trans-neuronal transport in vivo depends on synaptic contacts and strongly correlates with synaptic input strength. This experiment showed lower connectivity in SCZ iPSC-neurons, which also possessed fewer neurites and expressed lower levels of the synaptic marker PSD95 (postsynaptic density protein 95). Despite these deficits in connectivity, electrophysiological recordings and calcium transient imaging showed normal spontaneous activity in SCZ iPSC-neurons (Table 4).

Microarray analyses evidenced that close to 600 genes were differentially expressed in the neurons from patients with SCZ (271 upregulated, 325 downregulated) relative to controls [75]. Among these, one-quarter had been previously implicated in SCZ, either through GWAS or postmortem gene expression analysis. Gene ontology analysis detected significant alterations in genes that are involved in glutamate, cAMP, and WNT (Wingless) signaling; all of these pathways contribute to activity-dependent modulation of synaptic connections and LTP. The researchers also detected CNVs with weak effects on gene expression in SCZ iPSC-neurons, but none of the high penetrance CNVs associated with SCZ.

Interestingly, application of the atypical neuroleptic loxapine during the last three weeks of neuronal differentiation enhanced neuronal connectivity and glutamate receptor expression (i.e., *GRIK1*, *GRM7*, and *GRIN2A*) and improved the expression of genes that are involved in WNT and other signaling pathways (i.e., *WNT7A*, *TCF4*, *ADCY8*, and *PRKCA*) in SCZ iPSC-neurons [75]. Of cautionary note, structurally related antipsychotics failed to rescue the deficits in SCZ iPSC-neurons.

In light of the retained spontaneous activity in SCZ iPSC-neurons, Roussos et al. [76] moved on to investigate differences in activity dependent gene expression. Potassium chloride treatment was used for neuronal depolarization of iPSC-derived case/control forebrain neurons. Under the resting condition, RNA-sequencing (RNA-seq) evidenced 1669 differentially expressed genes (845 upregulated, 815 downregulated) in neurons from patients with SCZ relative to controls, which were enriched in pathways that were related to organization of the extracellular matrix (e.g., neural cell adhesion molecules, neuroligins, and neurexins, all of them with a role in cellular differentiation and migration) and calcium binding, which is in agreement with previous microarray analysis [75].

Furthermore, 1199 genes (595 upregulated, 604 downregulated) were differentially expressed in response to neuronal depolarization. Interestingly, depolarization of SCZ iPSC-derived forebrain neurons showed an attenuated transcriptional response with approximately 10-fold fewer genes relative to controls. Coexpression network analysis identified 15 modules, among which each two modules associated with diagnosis status and depolarization. Furthermore, SCZ GWAS risk genes [49] were strongly enriched in three of the four modules.

To investigate whether deficits in neuronal connectivity and activity-dependent transcription date back to early stages, Topol et al. [77] conducted RNA-seq profiling on NPCs from case/control iPSCs [75] and three additional controls. Among 848 significantly differentially expressed genes, they found a significant enrichment (3.6-fold) in WNT target genes in SCZ iPSC-derived NPCs relative to controls as well as in hedgehog, bone morphogenetic protein, and G-protein coupled signaling. Although a WNT responsive reporter assay showed enhanced activity in SCZ NPCs relative to controls, radial migration of NPCs from both groups appeared to be initially undistinguishable under in vitro conditions. However, in a separate study [70], the researchers could corroborate an impaired migration of SCZ iPSC-NPCs in three different test systems, but not in an unrelated wound healing assay, speaking against a general defect in cell motility. Consistent with these findings, they also found that cytoskeletal remodeling proteins (cofilins, CFL1 and CFL2 and profilins, PFN1 and PFN2) were up-regulated SCZ iPSC-NPCs [70].

Collectively, these iPSC-based case/control studies suggest perturbations in neurodevelopmental pathways as early as the NPC stage in SCZ (Table 4). These alterations seemed to affect NPC migration in vitro and they could predetermine lower neuronal connectivity and activity-dependent transcription at more mature stages that may be rescued by the application of neuroleptics.

Notably, changes in gene expression under resting and activated conditions in neuronal cells were enriched in GWAS variants [75,76], which thus contribute potentially to early neurodevelopmental alterations in SCZ. Intriguingly, normal spontaneous activity in SCZ iPSC-neurons is also consistent with the hypothesis that early neurodevelopmental changes may stay latent until called into operation at a later adolescent stage.

#### 1.5.2. Dopaminergic Neurons

Robicsek et al. [78] reprogrammed keratinocytes from three unrelated paranoid patients with familial SCZ and 2 independent healthy controls via a lentiviral vector. Single quality controlled clones (Table 1) were tested at different passages (p°9 vs. p°30); moreover, keratinocytes from one patient were reprogrammed again after one year.

Given well-known evidence for altered dopamine and/or glutamate signaling (see introduction), the researchers chose to study both type of neurons derived from SCZ iPSCs. Following neuronal differentiation by the dual-Smad inhibition protocol (Table 3), neuronal precursors from patients showed a protracted differentiation (larger cell size, higher PAX6, and lower Nestin expression) relative to controls and produced significant less dopaminergic neurons (β3-Tubulin/Tyrosine hydroxylase/Dopamine membrane transporter immunopositive cells) with underdeveloped neurites. In fact, none of the tyrosine hydroxylase-positive neurons expressed the dopamine membrane transporter, which is a specific marker for dopaminergic neurons. This deficit associated with a diminished release of dopamine, but not of serotonin, in patient relative to control cells.

In a parallel approach, Robicsek et al. [78] differentiated case/control iPSCs via intermediate embryoid body (EB) formation into forebrain glutamatergic neurons. Interestingly, patient derived iPSCs produced less β3-Tubulin-positive neurons with underdeveloped synapses and were devoid of the glutamatergic maturation marker TBR1 (T-box brain 1) relative to controls. 

Dopaminergic differentiation and cell function in SCZ iPSCs has been reinvestigated in two independent studies [79,80]. Hook and coworkers differentiated SCZ iPSCs that were obtained from Brennand et al. [75] into forebrain neurons. Mixed glutamatergic-GABAergic neuronal cultures were depolarized by potassium chloride treatment to measure activity-dependent secretion of catecholamines (dopamine, norepinephrine, and epinephrine) and several peptide neurotransmitters (dynorphin A and (Met)enkaphalin). Interestingly, basal and stimulated secretion of dopamine, norepinephrine and epinephrine was greater in SCZ iPSC-neurons relative to controls and concurred with an increased percentage of tyrosine hydroxylase-positive neurons (Table 4).

Given current limitations to produce strictly region-specific cell types, Hartley et al. [80] sought to clarify the identity of dopaminergic neurons that were derived from SCZ iPSCs [75] by assessing additionally the expression of the midbrain dopaminergic marker FOXA2 (forkhead box A2). For this purpose, the researchers differentiated case/control iPSCs via an intermediate step of EB formation into neuronal cells using a protocol closely related to the one of Robicsek et al. [78] and treated low passage NPCs additionally with a potent GSK3β inhibitor to prevent hypothalamic precursor formation (Table 3). While this approach led to an increased number of tyrosine hydroxylase and FOXA2-positive neurons, there were no significant differences between the case/control groups. 

Taken together, the reported differences in dopaminergic cell numbers and function in case/control studies point to a critical issue in tracing early neurodevelopment with patient-specific iPSCs: The use of clinical heterogeneous samples (paranoid SCZ [78] versus common SCZ [75]) and of different differentiation protocols. Robicsek et al. observed region- and cell-type specific neurodevelopment deficits in SCZ rather than a pan-neuronal failure in differentiation and maturation. Critically though, a reduced number of midbrain dopaminergic cells is at odds with the presumed hyperdopaminergic state in SCZ and it invites reflection on the regional identity of the dopaminergic neurons given that expression of tyrosine hydroxylase and of dopamine membrane transporter is widespread in the mammalian brain. In fact, Hook et al. reported that SCZ iPSC-forebrain neurons preferentially differentiated into and/or survived as tyrosine hydroxylase-positive neurons relative to controls. In light of these discrepancies, Hartley et al. used an optimized protocol for midbrain dopaminergic differentiation combined with the specific marker FOXA2 and detected no difference between case/control iPSCs. Hence, future studies are needed to develop refined differentiation protocols, highly informative markers, and stratified patient populations to resolve the issue of altered dopaminergic function across different brain regions during early neurodevelopment in SCZ.

#### 1.5.3. Hippocampus

Perturbations in hippocampal neurogenesis are thought to contribute to cognitive deficits in SCZ among other psychiatric diseases [88]. Yu et al. [81] investigated case/control iPSCs [75] by two different differentiation protocols to obtain neurons expressing PROX1 (Prospero-related homeobox 1) and TBR1; two markers expressed in mature dentate gyrus (DG) granule neurons. 

Free-floating EBs were treated with anticaudalizing factors to enrich for telencephalic neural precursors (Table 3). Thereafter, an inhibitor of the sonic hedgehog (SHH) pathway was applied to obtain dorsal forebrain progenitors, followed by treatment with a wingless agonist and brain derived neurotrophic factor; both of these factors are important for the maintenance of hippocampal progenitors and their differentiation into DG granule neurons (Figure 1). At various time points, EBs expressed key regulators of hippocampal development in a manner closely mimicking their sequential expression pattern in vivo. In particular, about one-third (~32%) of the mature EBs expressed the specific marker gene PROX1 when compared to only few cells (~5%) under the untreated condition. Finally, EBs were dissociated and co-cultured with human hippocampal astrocytes to promote neural network formation and maturation. 

In a modified version of this protocol [81], EBs that were treated with anticaudalizing factors (Table 3) were platted as monolayers to allow for rosette formation, a structure resembling the early neural tube (Figure 1). NPCs were enriched by manual dissection and differentiated into DG granule neurons, as described above. About 70% of the neurons were immunopositive for PROX1, more than 85% for the vesicular glutamate transporter, and less than 15% for GABA.

Following four weeks of differentiation, PROX1-positive neurons were largely active with spontaneous bursts of action potentials and postsynaptic currents and showed normal currents and action potentials in response to electrical stimulation. Calcium transients increased across differentiation and neuronal maturation consistent with the formation of functional neural networks. Furthermore, early NMDA receptor-mediated inhibition passed into late AMPA mediated inhibition tracing granule neuron maturation and circuit integration in vivo [89]. Lastly, iPSC-hippocampal neurons integrated efficiently into the postnatal maturating hippocampus of mice, 40% were PROX1-positive, and sent extensive processes to the CA3 region along the mossy fiber path.

Tracing early hippocampal differentiation and function in case/control iPSC, Yu et al. detected a reduced expression of NEUROD1 (neuronal differentiation 1), PROX1, TBR1, and FOXG1 (Forkhead Box G1, a regulator of NPC proliferation and survival of newborn DG neurons) during the differentiation of SCZ iPSCs. This delayed differentiation correlated with significantly less calcium transients indicating that deficits in neural differentiation contributed to impaired neuronal network activity. However, PROX1-positive granule cells from SCZ iPSCs retained basic neuronal characteristics of Na^+^/K^+^ currents and evoked action potentials. By contrast, spontaneous neurotransmitter release was significantly reduced in the frequency and amplitude of spontaneous excitatory postsynaptic currents in neurons from patients with SCZ relative to controls.

Most recently, Sarkar et al. [82] refined Yu’s protocol [81] for the generation of hippocampal DG-CA3 circuits from case/control iPSCs [75]. Differentiated neurons were mostly glutamatergic (>90%) and expressed genes specific to the CA3 and the broader CA3 field (i.e., including both CA2 and CA3). By contrast, markers of the DG were poorly present in six-week-old CA3 cell populations. Single cell transcriptomics of two-week-old CA3 cells showed the presence of immature, neuronal, and known hippocampal markers with a role in cell morphogenesis, synaptic transmission, and neuron migration. iPSC-CA3 neurons showed robust Na^+^ and K^+^ currents, spontaneous and evoked action potentials, and spontaneous excitatory postsynaptic currents. In support of a functional network, CA3 neurons formed dense connections and exhibited robust electrical activity, as evidenced by rabies virus spreading [75] and multiple-electrode arrays (MEA). By contrast, human iPSC-derived DG neurons were largely inactive and unconnected. Interestingly though, co-culturing CA3 and DG neurons stimulated the formation of a DG-CA3 circuit in the absence of afferent activity and exogenous axon guidance cues. Importantly, spontaneous spike and network bursts in SCZ-DG-CA3 co-cultures were reduced at six-weeks relative to controls although both co-cultures contained similar numbers of inhibitory interneurons (<5%). Since network activities were undistinguishable at earlier stages of development, deficits between cases and controls seemed to evolve during maturation.

In sum, iPSC-based case/control studies showed that delayed hippocampal differentiation of SCZ NPCs led to impaired network connectivity and activity and thus resembled the results on SCZ-iPSC derived forebrain neurons [75,77]. Additionally, iPSC-derived DG-CA3 circuits revealed deficits in spontaneous and evoked electrical activity during maturation, but not at earlier stages of neurodevelopment. It is interesting to note that early developmental delay comprised network connectivity and activity when the hippocampal cells were grown in the absence [81] or the presence [82] of a CA3-DG circuit. This indicates that proper innervation did not rescue genetically driven deficits in early development and maturation of SCZ-iPSC derived hippocampal neurons.

#### 1.5.4. Oxidative Stress in SCZ iPSCs

**Figure 3:**
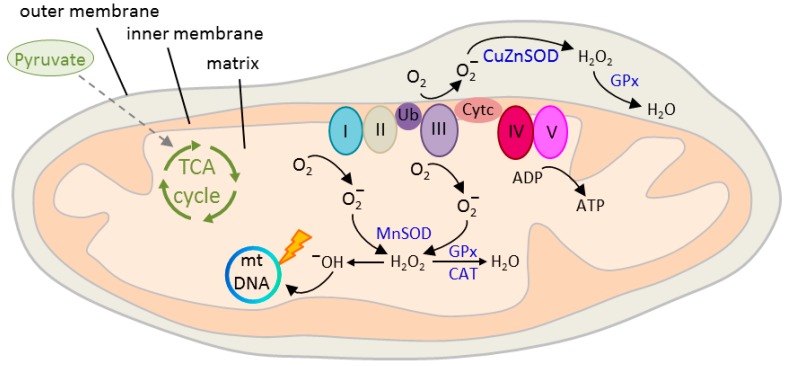
Structure and function of the mitochondrion. Outer and inner double-layered membranes enclose the intermembrane space, the cristae space formed by infoldings of the inner membrane, and the matrix as the space within the inner membrane. The matrix hosts the circular mitochondrial DNA (mtDNA) and a large number of enzymes catalyzing various biochemical reactions. Blood-borne glucose is metabolized intracellularly to pyruvate and then to acetyl-CoA, the only substrate fueling the tricarboxylic acid (TCA) cycle. Oxidation of pyruvate produces reduced cofactors that transfer free electrons to the electron transport chain (ETC) hosted at the inner mitochondrial membrane. The ETC consists of complex I to V that build an electrochemical gradient (∆φ_m_) across the inner mitochondrial membrane driving the synthesis of adenosine triphosphate (ATP). Electron leakage mainly at complex I and III (0.1 to 2%) reduces prematurely oxygen to superoxide anion (O_2_^−^) that is converted by mitochondrial superoxide dismutase MnSOD and CuZnSOD to hydrogen peroxide (H_2_O_2_) in the matrix or intermembrane space, respectively. Thereafter, H_2_O_2_ is oxidized to highly reactive hydroxyl (OH^−^) free radicals that cause mtDNA damage, reduced transcription, and oxidation of ETC proteins and membrane lipids. Radical production is counteracted by enzymatic defense mechanisms, including glutathione peroxidase (GPx) and catalase (CAT) that detoxify H_2_O_2_ into water. Ub = ubiquinone, also known as Coenzyme Q10; Cyt c = cytochrome c.

Neurons are responsible for most (≈80–90%) of the brain’s energy demand [90] that is largely satisfied (>90%) by the mitochondrial tricarboxylic acid (TCA) cycle (also referred to as citric acid cycle or Krebs cycle). The TCA cycle oxidizes acetyl-CoA to carbon dioxide and produces reduced cofactors that are a source of electrons for the electron transport chain (ETC, also referred to as respiratory chain) (Figure 3). The ETC drives the synthesis of adenosine triphosphate (ATP) and it is also the major source (≈90%) [91] of deleterious reactive oxygen species (ROS) production: Leakage of electrons leads to the reduction of oxygen to superoxide anion (O_2_^−^) [92] that is metabolized to hydroxyl (OH^−^) free radicals (Figure 3). These highly reactive molecules cause serious damage to nucleic acids, proteins, and lipids [93].

The human fetal brain is particularly vulnerable to oxidative damage given its high oxygen consumption relative to the adult brain and its high basal levels of ROS production. The developing brain contains also high concentrations of polyunsaturated fatty-acids that are a preferred substrate in lipid peroxidation, while antioxidant defense mechanisms are still less developed as compared to the adult [94]. Epidemiological risk factors in SCZ such as gestational complications, malnutrition, and infections associate with an increased oxidative load [95,96,97] that can lead to early neurodevelopment perturbations. Beyond prenatal life, studies on postmortem brain and peripheral tissues from patients with SCZ have pointed to altered redox regulation that seems to reflect poorly understood genetic risk factors in this disease [98,99]. 

Paulsen and coworkers [83] reprogrammed skin fibroblasts from a clozapine resistant patient with SCZ and an age-matched control into iPSCs (Table 1). Following retinoic acid-induced differentiation (Table 3), dopaminergic, cholinergic and serotonergic markers, voltage-activated channels, and functional synapses were equally expressed in patient and control neurons. By contrast, extra-mitochondrial oxygen consumption was about two-fold higher in differentiated SCZ iPSCs relative to controls, while no differences were detected in the undifferentiated iPSCs or the parental fibroblasts. Oxygen flow in the SCZ iPSC neurons correlated to higher ROS levels that were reversed by treatment with the mood stabilizer valproic acid, a known protectant against oxidative stress and an adjunctive medication for SCZ. The researchers further showed that the trace elements potassium and zinc were elevated in SCZ iPSC neurons and were normalized by valproate treatment [100].

Mitochondrial function is regulated in response to a cell’s metabolic demand, but also through neurotransmission, and plays a critical role in development, synapse formation, and plasticity of spines and synapses [99,101,102]. As discussed before, Robicsek and coworkers [78] reported that SCZ iPSCs were impaired in dopaminergic differentiation and glutamatergic maturation. Interestingly, the researchers further found an abnormal expression of three subunits of complex I, a multiprotein complex of the ETC (Figure 3) that correlated with a decrease in mitochondrial membrane potential (∆φ_m_) in undifferentiated SCZ iPSCs and during differentiation into dopaminergic and glutamatergic neurons. Mitochondrial respiration and sensitivity to dopamine-induced inhibition were impaired in keratinocytes, iPSCs, and neuronal differentiated cells derived from patients with SCZ pointing to a basic defect extending across-cell types and developmental stages. This deficit concurred with an uneven cellular distribution of the mitochondria that is consistent with an impaired function. In addition, mitochondrial network connectivity, an indicator of mitochondrial activity, was impaired in dopaminergic, but not in glutamatergic neurons. 

A follow up study by Brennand and coworkers [70] lends further support to altered redox mechanisms in SCZ. The researchers conducted genome-wide expression profiling and quantitative proteomic mass spectrometry on case/control NPCs [75,77]. Microarray profiles from forebrain NPCs and six-week-old neurons evidenced 481 significantly differentially expressed genes (134 upregulated and 347 downregulated). Most of the changes from the NPC state were retained in mature neurons, indicating that they precede neuronal differentiation. Bioinformatic analysis on SCZ iPSC-NPCs identified alterations in gene modules regulating neuron differentiation, neuronal migration, glutamate receptor signaling, synaptic vesicle functions, and cellular adhesion.

Interestingly, mass spectrometry additionally detected the upregulation of oxidative stress pathways in SCZ iPSC-NPCs. Top ranked proteins included thioredoxin, an antioxidant cofactor, and thioredoxin-like or thioredoxin domain containing proteins [70].

Mitochondrial function and oxidative stress are tightly interwoven: Mitochondrial dysfunction enhances ROS formation, and vice versa, increased ROS formation reduces mitochondrial membrane potential (MMP) through mitochondrial damage. Notably, SCZ iPSC-NPCs showed significantly decreased MMP, indicative of oxidative stress, relative to controls [70]. Morphologically, SCZ NPC-mitochondria were smaller in size, less connected, and less densely packed around the nucleus relative to control NPCs. Furthermore, protein oxidation and DNA damage was enhanced in SCZ iPSC-NPCs relative to controls. 

To assess the cause-effect relationship for mitochondrial dysfunction in SCZ, Robicek et al. [84] most recently investigated mitochondria transfer to SCZ-iPSC derived neurons [78]. Several studies have shown that mitochondria can transfer between cells via various contact modes, comprising cell fusion, junction, and tunneling nanotube formation [103]. In support of these findings, Robicek et al. observed that in vitro isolated active normal mitochondria (IAN-MIT) can enter various cell types without any manipulation and stay active. IAN-MIT transfer into lymphoblasts from a patient with SCZ normalized impaired basal respiration, enhanced dopamine inhibitory effect on basal respiration and membrane potential, and reversed uneven cellular distribution of the mitochondria. By contrast, reduced network connectivity remained unaffected. In SCZ iPSCs, IAN-MIT transfer likewise recovered lastingly membrane potential and mitochondria distribution, resulting in improved glutamatergic differentiation (increased expression of the neuronal marker β3-tubulin, the glutamatergic maturation marker Tbr1, and of synapsin). Furthermore, in rats with a history of prenatal immune activation, a translational model for SCZ, intra-prefrontal cortex injection of IAN-MIT prevented mitochondrial membrane reductions and attentional deficits in adolescence.

Collectively, these studies showed perturbed mitochondrial respiration (reduced membrane potential) and morphology (smaller sized, less-connected mitochondria) concomitant with signs of increased oxidative damage (upregulations of thioredoxin, protein oxidation, and DNA damage) in SCZ iPSC-NPCs and neurons relative to controls. Furthermore, basic defects in SCZ (e.g., perturbed respiration) seemed to exist across different developmental stages, while more subtle defects (e.g., altered network connectivity) seemed to unfold during the distinct stages of development and differentiation. Intriguingly, mitochondrial transfer improved mitochondrial dysfunction and glutamatergic differentiation of SCZ iPSCs and prevented behavioral deficits in an animal model of this disorder. These results indicate that mitochondrial transfer may benefit diseases with bioenergetic and neurodevelopmental abnormalities, such as SCZ. 

#### 1.5.5. MicroRNAs in SCZ iPSCs

miRNAs are noncoding RNAs [104] of ~70 nucleotides in size (pri-miRNAs) that are cleaved by a nuclear protein complex consisting of DGCR8 and DROSHA into precursor RNAs (pre-miRNAs). In the cytoplasm, pre-miRNAs are further cleaved by DICER to generate single stranded ~22 nucleotide mature miRNAs. Upon incorporation into the RNA induced silencing complex (RISC), they target through a 6- to 8-base pair complementary ‘seed region’ (typically in the 3′untranslated region) one or more mRNAs. miRNAs down-regulate gene expression with each miRNA potentially controlling up to hundreds of downstream targets [104]. In the developing human brain, miRNAs are highly expressed and regulate neural lineage and cell fate decisions, differentiation, and neuronal maturation [105,106]. In the adult brain, miRNAs also regulate neuroplasticity. Altered miRNA expression profiles have been observed in SCZ, autism, and major depression [107]. For example, miRNA-137 (miR-137) maps to a risk locus of SCZ [58,108] and it may regulate disease-relevant genes, like *CACNA1C* (calcium channel, voltage-dependent, l-type, alpha-1C subunit) or *TCF4* (transcription factor 4) [109,110]. 

To assess the role of *MIR137* SNP genotypes, Siegert and coworkers [85] chose six human fibroblast lines that were homozygous for either the common, major allele, or for the rare, minor allele for all four disease-associated SNPs. The fibroblasts were directly converted into early neuron-like cells (Table 1 and Table 3) and further FACS-purified four-weeks post-transduction. Interestingly, miR-137 expression was significantly increased in early neuron-like cells derived from the minor allele group relative to the major allele group. By contrast, expression levels in the parental fibroblasts did not differ between the alleles indicating that the minor allele SNPs operated in early neurons only. Bioinformatic analysis predicted 21 potential miR-137 target genes with well-known roles in presynaptic vesicle trafficking. Consistent with this prediction, enhanced miR-137 levels in induced early neurons reduced vesicle trafficking following neuronal excitation. 

Moving beyond cell culture, Siegert et al. showed for a subset of the verified target genes downregulation in the synaptosomal fraction of miR-137 transduced DG neurons from adult mice. This downregulation reduced vesicle formation and LTP, a proxy to neuronal plasticity, in presynaptic terminals of the mossy fiber. Contrariwise, the sequestration of miR-137 expression induced miR-137 target genes in induced early neurons carrying the minor SCZ risk allele and enhanced stimulus-dependent vesicle release in vitro and in vivo. On a cautionary note, the alleles actually mediating disease risk and the extent to which single disease-associated SNP relate to SCZ remained unanswered by these experiments. 

In an elegant study, Forrest and coworkers [55] have advanced this issue by determining the genome-wide landscape of open chromatin regions (OCRs) in iPSC-derived neurons. ATAC-sequencing (Assay for Transposase-Accessible Chromatin) showed that OCRs change dynamically during early neuronal differentiation and are characteristic of distinct stages. Focusing on the *MIR137* locus, the researchers narrowed down common risk variants to a potentially functional SNP (rs1198588) in a neuronal OCR. On cautionary note, only a subset of the GWAS SNPs was present in neuronal OCRs, allowing for putatively functional SNPs to be prioritized. 

Isogenic SCZ iPSC lines differing solely at the predicted functional GWAS risk SNP were generated by CRISPR/Cas9 editing. Modifying this risk SNP to a non-risk-allele affected chromatin dynamics at the *MIR137* locus and led to increased miR-137 expression during induced differentiation of glutamatergic forebrain neurons concomitant with a reduced maturation of the dendritic arbor. Conversely, presence of the GWAS SNP risk reduced miR-137 expression and enhanced the maturation of glutamatergic forebrain neurons. 

Moving beyond single candidates, Topol et al. [86] investigated miRNA expression in case/control NPCs [70] and a new cohort of each 10 patients with childhood onset SCZ and controls (Table 1). Among 800 miRNAs that were detected by digital expression profiling (Nanostring), miR-9, a regulator of neurogenesis in NSC [111], was the most abundant and the most downregulated miRNA in NPCs. Subsequent analysis of the adult cohort, for which mRNA expression profiles from both NPCs and neuronal cells were already available [70], showed that known miR-9 target genes were significantly enriched (*n* = 84) among differentially expressed genes (56% upregulated, 44% downregulated).

Reduced miR-9 activity in SCZ iPSC-NPCs subsisted in 1- and 2-week old neurons, but not in mature neurons, and correlated with reduced outgrowth migration of neurospheres. Stable miR-9 overexpression in SCZ iPSC-NPCs, but not in controls, improved the total radial migration, whereas transient knockdown of miR-9 in controls decreased total radial migration. Interestingly, differentially expressed genes from the miR-9 perturbation experiment were significantly overrepresented among differentially expressed genes from the SCZ iPSC-NPCs signature and provide experimental evidence for the prediction that miR-9 contributes to the differences between SCZ and control NPCs. Among the shared differentially expressed genes, only few matched direct miR-9 targets, while most of them appeared to be upregulated indirectly and consisted of genes relevant to the function of the plasma membrane, cell adhesion, and extracellular matrix.

Topol et al. [86] investigated also the effect of miR-9 overexpression on global protein translation in case/control iPSC-NPCs and observed that pathways regulating actin cytoskeleton, protein localization, and RNA processing were most affected in either group. By integrating global RNA and proteomic analyses of miR-9 perturbations, the researchers concluded that small changes in miR-9 targets might ensue from reduced miR-9 levels in a subset of SCZ iPSC-NPCs. Compatible with this view, previous SCZ GWAS gene-set enrichment analysis [49] has found an enrichment on predicted miR-9 targets among SCZ-associated genes [112]. Together, these findings suggest that genetic variants in both miR-9 and its targets confer an increased risk of SCZ. 

Focusing on early steps of neuronal differentiation, Narla et al. [87] recently analyzed case/control iPSCs [75] for changes in the miRNA transcriptome. NPCs were differentiated for two days in neuronal differentiation media to obtain ‘neuron committed cells’ (NCCs) as a proxy to incipient neural development (Table 2 and Table 3).

The researchers identified among 440 detectable miRNAs 16 transcripts that were upregulated in SCZ relative to controls. These differentially expressed miRNAs were predicted to target over 400 mRNAs in a largely overlapping mode. Upregulated transcripts included miR-218 [113], miR-132 [114], miR-134 [115,116], and miR-17 [117], all of these have been previously implicated in SCZ and are thought to contribute to neural development and cell motility. Interestingly, differentially expressed miRNAs related rather weakly with the expression of their target mRNAs in SCZ, but not in control NCCs indicating a reduced regulatory capacity of miRNAs in diseased ‘neuron committed cells’.

Taken together, case/control iPSC studies in SCZ support perturbed miRNA expression and/or function at the level of single genes or complex networks. Different alleles of the SCZ risk gene *MIR137* associate with different levels of miR-137 expression with the direction of the effects depending critically on the genetic background and nature of the experimental system. In any case, any expression differences in miR-137 affect critically neuronal plasticity of the early neurodevelopmental stages in vitro and mature neurons in vivo. Relatedly, reduced expression of miR-9 in SCZ iPSC-NPCs impairs cell migration via small perturbations of a large network of target genes that are enriched for SCZ risk loci. Finally, miRNA networks enriched in SCZ risk miRNAs may be disrupted at a global scale in early neural cells from SCZ iPSCs due to an impaired regulation of their target genes. In brief, miRNAs seem to contribute crucially to perturbed early neurodevelopment in SCZ.

## 2. Future Directions

The advent of iPSC technology has opened unprecedented opportunities for the study of living disease-relevant cells from patients with idiopathic SCZ. Case/control studies highlight perturbed neuronal development, differentiation, and maturation in SCZ: Neurons show decreased neurite numbers [75], reduced synaptic maturation and activity [75,78,81], and impaired activity-dependent transcription [76]. Furthermore, SCZ NPCs show reduced migration [70], perturbed WNT signaling [77], increased oxidative stress [70,78,83], and changes in miRNA expression and function [86,87,118]. Some of these processes may recapitulate processes from the diseased brain and were ameliorated by pharmacological treatments [75,100]. Overall, SCZ-iPSC studies provide strong experimental support for previous epidemiological, clinical, genetic, and circumstantial evidence on a neurodevelopmental component in SCZ. Notwithstanding this remarkable progress, we next discuss some caveats that may confound the current findings and require further improvements on the generation and design of patient-specific iPSC studies.

### 2.1. High Resolution Karyotype Maps

A critical issue in case/control studies is the occurrence of “random” mutations during the reprogramming process and/or prolonged in vitro culture and passage. In any case, non-integrating, so-called “foot-print free”, reprogramming techniques (i.e., Sendai virus, episomal, and mRNA transfection) are preferred to retro- or lenti-viral vectors that via integration into the host genome may confound molecular and cellular phenotypes (Table 1). “Foot-print” techniques have been readily accepted over the last years; however, they are no universal remedy. Traditional G-banding detects only large aneuploidies over 5 MB in size. By contrast, while using an SNP array system with an average genomic resolution of 43 KB, Schlaeger et al. [119] detected the highest aneuploidy for retroviral (13.5) and episomal (11.5%) derived iPSCs, in-between aneuploidy for lentiviral (4.5%) and Sendai virus (4.6%) derived iPSCs, and lowest aneuploidy for RNA (2.3%) derived iPSCs. To elucidate the origin of these aberrations, Kwon et al. [120] as compared the mutational load of clonal fibroblast lines and iPSC lines that are produced from the same fibroblast. Whole exome sequencing showed that clonal fibroblasts and iPSCs contained a similar number of mutations with more than 90% of them preexisting randomly in small subsets of the parental unselected fibroblast population as evidenced by deep, targeted resequencing. While there is plenty of evidence that common genetic variation [121,122,123,124,125,126], and so much more chromosomal aneuploidy, cause molecular heterogeneity in iPSCs, none of the present iPSC-based studies on idiopathic SCZ (Table 1) has applied donor-matched digital (i.e., SNP-based) karyotype maps to detect chromosomal anomalies more precisely. Chromosomal anomalies extent also to copy number alterations, as shown by high resolution karyotype mapping (detection limit > 200 KB) of donor-matched samples [124].

Digital karyotyping benefits other aspects of iPSC quality control additionally: Under in vitro selection pressure, long-term passaged iPSC lines are prone to acquire growth-promoting mutations, which are likely to confound differentiation analysis. Moreover, the misassignment of iPSC samples can occur at any stage on the tedious way from reprogramming to neuronal differentiation. The authenticity of iPSC lines should be controlled on a routine basis; particularly in those cases where iPSC lines are shared across multiple studies and between different laboratories (see Table 1). Relatedly, digital karyotyping enables the verification of familial relationships. In brief, digital karyotyping is an important tool to ensure and maintain comparability in iPSC-based case/control studies.

### 2.2. Assessing Differentiation Capacity

A large body of studies has helped to optimize the generation and functional characterization of iPSCs [67,127]. Most SCZ iPSCs were evaluated for functional pluripotency by EB or teratoma formation, which confirmed that case and control donor cells were equally efficiently reprogrammed. Still, none of the applied tests (Table 1), nor any currently available test, can predict the capacity for differentiation towards distinct neuronal tissues and cell types that are at the focus of case/control studies. Differences in the (neural) differentiation capacity of human embryonic stem cells and iPSCs from healthy donors exist [128,129,130,131] and they are driven by differences in genotype, expression profiles, and epigenetic state of individual iPSC lines that are frequently randomly distributed unrelated to disease state [132]. A recent large scale iPSC study (*n* = 317) on healthy donors has found that ~49% of the genome-wide expression variability in the pluripotent state relates to variation across individuals and it can be ascribed in part to differences in expression quantitative trail loci [123]. The remaining 50% are likely due to random fluctuation in epigenetic state present, even in distinct clones from the same iPSC line. Epigenetic variability detected within and across individuals reflects in part the activity of Polycomb proteins in agreement with their important role during (neuro) development [133]. In consequence, donor-specific variability can critically bias neuronal differentiation propensity independent of disease status in case/control studies (Table 2 and Table 3).

At this time, there is no single criterion to distinguish between the impact of normal and disease-related forms of variability on early neurodevelopment in vitro. Precautions include the use of single iPSC clones at different passage numbers [78] or of multiple iPSC clones per donor at similar passage numbers (Table 1) to reduce culture-dependent effects. Similarly, increased donor cohort sizes of well-stratified patient populations (see below) are critical to distinguish truly disease associated changes in (endo-) phenotypes from random line and culture artifacts. Moreover, the use of different or unrelated differentiation protocols may inform on the general deficits in proliferation and differentiation [81]. Likewise, differences between case/control iPSCs appear to be more important if cell-type specific [78] and present in disease “relevant” contexts. This raises concerns about how to define “relevance” in light of our limited understanding of the biology underlying SCZ. In fact, deficits across tissues and cell types may manifest only through pathways enacting the function of vulnerable templates [76]. As of yet, none of these precautions will provide a definite answer how to quantify differentiation capacity robustly.

On top of these concerns, neural differentiation protocols are cumbersome, poorly scalable, and introduce through the production of heterogeneous cell-populations that can vary even between individual lines of the same donor additional variability [134]. Although well-known, few studies sought to systematically determine the impact of these factors on quantifiable measures. In a recent large scale study, Schwartzentruber et al. [135] differentiated iPSCs (*n* = 123) derived from healthy donors into sensory neurons and found that sample-to-sample variability in the gene expression in iPSC derived cells clearly exceeded the one from in vivo dorsal root ganglia, whereby genes with an important role to this cell type were among the most variables. Crucially, levels of variation for donor and reprogramming (23.2% in aggregate) were comparable to those from the neuron differentiation batch (24.7%), which related in major part to the existence of varying mixture of cell types across differentiation [135]. 

It is also important to note that current differentiation protocols applied to iPSC-based case/control studies on SCZ neglect the three-dimensional organization of the human brain and well-known structure-function relationships [136]. Therefore, differences in the responses of types or locations of iPSC-derived neurons from patients with SCZ could reflect the fact of regional specificity seen in imaging/computational models of patients [137]. Recent progress on the generation of region-specific brain organoids [138,139] is expected to address this issue at least in part. 

All in all, these limitations call for refined neuronal differentiation protocols to reduce intra- and interdonor cellular heterogeneity and to improve structure-function relationships in order to assess neuronal differentiation capacity reliably. Right now, multiple karyotype-controlled iPSC lines per donor should be investigated to identify those yielding more homogeneous neuronal cell types, while the implementation of automated cell sorting systems and single cell sequencing techniques can improve the resolution of cell-autonomous effects. 

### 2.3. Modeling of Polygenic Disorders

iPSC-based disease modeling has gained momentum from the field of Mendelian disorders, which require only small cohort sizes, owing to the presence of rare, highly penetrant genetic variants that associate with distinct cellular and molecular effects. By contrast, polygenic disorders harbor different arrangements of numerous (non-) coding variants of small effect size that may result in less clear-cut or more complex phenotypes. Notwithstanding this reservation, current iPSC studies on SCZ have succeeded in the identification of a number of cellular and molecular anomalies (Table 4). Critically though, many of the studies that are reviewed here relied on the same or only slightly different sets of patients with SCZ and control iPSC lines (Table 1). This raises the issue to what degree these findings can be generalized or specify instead a subset of patients. In any case, careful selection of controls is by no means less important than the one of patients and genetically-informed selection [140] for SCZ iPSC studies may benefit reducing cellular heterogeneity. While most researchers agree that cohort size should be enlarged, the size of cohorts needed is still a matter of uncertainty. Schwartzentruber et al. [135] have cast some light on this issue: Using a large collection of iPSCs (*n* = 123), they succeeded in the identification of thousands of quantitative trait loci regulating gene expression, chromatin accessibility, and RNA splicing during neuronal differentiation. Based on these findings, iPSCs from 20–80 donors appear to be sufficient to detect the effects of common regulatory variants of moderate to large effects sizes. Although larger samples sizes are always beneficial to identify disease-associated traits, there are limitations as to how many iPSC lines can be realistically analyzed in a typical laboratory setting. A potential solution to this conundrum may be to stratify patient samples based on their genetics (e.g., presence of a rare mutation) to identify the SCZ subtypes with more penetrate phenotypes or the use of high throughput technologies. While not written in stone, these guidelines can substantially improve the quality of iPSC studies on idiopathic SCZ we should look for in the future.

Overall, SCZ remains a global challenge to mental health with more affected people every day. The need to understand SCZ pathology persists despite a century of research and therapeutic progress. The transformative discovery of iPSCs [7] has widely opened the door for new translational strategies to trace the early neurodevelopment of SCZ in vitro. iPSC-based studies on patients with SCZ do not recapitulate the complex cellular and spatio-temporal phenotypes from the perinatal and adult brain, nor do they recreate clinical symptoms of adolescents or adults with SCZ in a dish. However, they allow for the study of a human-relevant disease in a dish to a certain degree and empower us to carry out quantifiable measures of neuronal morphology, function, electrophysiology, connectivity, and gene expression across embryonic and fetal brain development. All in all, the generation of living neurons from patients with SCZ can transform our mindscape of this disease through the identification of perturbed molecular networks and of targets for new, tailored therapies [141].

## Figures and Tables

**Table 1:** Methods for Schizophrenia-induced pluripotent stem cell (SCZ-iPSC) generation and quality control.

Ref.	Source	Factors	Met	N°	Auth	Karyo	Pluripotency
[75]	fibroblast	OKSML	LV	≥5	nd	G-B	ICC, Tera
[76]	[75]	OKSML	LV	≥5	nd	G-B	ICC, Tera
[77]	[75]	OKSML	LV	≥5	nd	G-B	ICC, Tera
[78]	keratinocyte	OKSM	LV	1	nd	G-B	ICC, PCR, PluriTest, EB
[79]	[75]	OKSML	LV	≥5	nd	G-B	ICC, Tera
[80]	[75]	OKSML	LV	≥5	nd	G-B	ICC, Tera
[81]	[75]	OKSML	LV	≥5	nd	G-B	ICC, Tera
[82]	[75]	OKSML	LV	≥5	nd	G-B	ICC, Tera
[83]	fibroblast	OKSM	RV	2	nd	G-B	ICC, PCR, EB
[70]	[75,77]	OKSML	LV	≥5	nd	G-B	ICC, Tera
[84]	[78]	OKSM	LV	1	nd	G-B	ICC, PCR, PluriTest, EB
[85]	fibroblast	APM	LV	na	na	na	ICC
[55]	fibroblast	OKSM	LV	dns	nd	G-B	ICC, PluriTest, EB
[86]	[75]	OKSML	LV	≥5	nd	G-B	ICC, Tera
	fibroblast	OKSM	Sen	2–3	nd	G-B	PCR, FACS
[87]	[75]	OKSML	LV	≥5	nd	G-B	ICC, Tera

Abbreviations are: APM, reprogramming factors ASCL1, POU3F2, MYT1L; Auth, authentication; EB, embryoid body formation combined with ICC; dns, data not shown; Epi, episomal plasmid; G-B, chromosomal G-banding; ICC, immunocytochemistry; Karyo, karyotype; LV, lentiviral transduction; Met, methods; N°, numbers of independent clones per donor; na, non-applicable; nd, not determined; OKSM, reprogramming factors OCT4, KLF4, SOX2, MYC, OKSML plus Lin28; PCR, quantitative reversed transcribed polymerase chain reaction; PluriTest, a bioinformatic tool to asses pluripotency; Sen, Sendai virus; Ref, reference; RV, retroviral; Tera, teratoma formation.

**Table 2:** Study design, cellular model, and neuronal cell types.

Ref.	Study Design	SCZ vs. Controls	Model	Major Cell Type(s)
[75]	Familial SCZ	4 vs. 3	iPSC	Forebrain, mixed glutamatergic-GABAergic neurons and NPCs
[76]	[75]	4 vs. 3	iPSC	Forebrain, mixed glutamatergic- GABAergic neurons
[77]	[75]	4 vs. 6	iPSC	Forebrain NPCs
[78]	Paranoid SCZ	3 vs. 2	iPSC	Midbrain dopaminergic and forebrain glutamatergic neurons
[79]	[75]	4 vs. 3	iPSC	Forebrain, mixed glutamatergic-GABAergic neurons
[80]	[75]	4 vs. 3	iPSC	FOXA2-positive midbrain dopaminergic neurons
[81]	[75]	4 vs. 3	iPSC	Dentate gyrus-like granule neurons
[82]	[75]	4 vs. 3	iPSC	CA3 and dentate gyrus neurons
[83]	CR-SCZ	1 vs. 1	iPSC	Retinoic acid-induced NPCs
[70]	[75]	4 vs. 6	iPSC	Forebrain NPCs
[84]	[78]	1 vs. 1	iPSC	Forebrain glutamatergic neurons
[85]	*MIR137* risk alleles	3 mar vs. 3 mir	iNLC	Early neuron-like cells FACS-purified
[55]	*MIR137* risk SNP	2 vs. 1 vs. 2 ig	iPSC	Induced forebrain glutamatergic neurons
[86]	[75]	4 vs. 6	iPSC	Forebrain NPCs
	Childhood-onset SCZ	10 vs. 10	iPSC	Forebrain NPCs
[87]	[75]	3 vs. 3	iPSC	Neuron committed forebrain NPCs

Abbreviations are: CR-SCZ, Clozapin resistant patient with SCZ; FACS, fluorescence activated cell sorting; ig, isogenic cell line; iNLC, induced neuron-like cell; iPSC, induced pluripotent stem cell; mar, major risk allele; mir, minor risk allele; NPC, neural progenitor cell.

**Table 3:** Major differentiation methods.

Ref.	Neural Induction	Patterning/Neural Progenitor Cells → Neural Cells
[75]	EB-/rosette formation	N2, B27-RA, FGF2 → N2, B27-RA, BDNF, GDNF, cAMP, AA
[76]	EB-/rosette formation	N2, B27-RA, FGF2 → N2, B27-RA, BDNF, GDNF, cAMP, AA
[77]	EB-/rosette formation	N2, B27-RA, FGF2
[78]	Nog, SB431542	SHH → SHH, FGF8, BDNF, AA
[79]	EB-/rosette formation	N2, B27-RA, FGF2 → N2, B27-RA, BDNF, GDNF, cAMP, AA
[80]	LDN193189, SB431542	SHH8 + FGF8 → BDNF, GDNF, cAMP, AA SHH8 + FGF8, CHIR99021 → BDNF, GDNF, cAMP, AA
[81]	DKK1, SB431542, Nog, Cyc	N2, B27, FGF2 → N2, B27, BDNF, Wnt3a, cAMP, AA
[82]	DKK1, SB431542, Nog, Cyc	N2, B27, FGF2 → N2, B27, BDNF, Wnt3a, cAMP, AA
[83]	Nog, bFGF	RA → FGF2
[70]	EB-/rosette formation	N2, B27-RA, FGF2
[84]	Nog, SB431542	SHH → SHH, FGF8, BDNF, AA
[85]	Lentiviral transduction	N2, bFGF
[55]	Dorsomorphin or Nog, SB431542	N2, B27 or B27-RA, FGF2
[86]	LDN193189, SB431542	N2, B27-RA, FGF2
[87]	EB-/rosette formation	N2, B27-RA, FGF2 → N2, B27-RA, BDNF, GDNF, cAMP, AA

Abbreviations are: AA, ascorbic acid; BDNF, brain derived neurotrophic factor; cAMP, cyclic adenosine monophosphate; Cyc, cyclopamine; DKK1, Dickkopf-related protein 1; EB, embryoid body; GDNF, glial cell derived neurotrophic factor; FGF, fibroblast growth factor; Nog, Noggin; RA, retinoic acid; SHH, sonic hedgehog; Wnt, wingless.

**Table 4:** Major analytical methods and findings in iPSC-based case/control studies in SCZ.

Ref.	Major Methods	Major Findings in SCZ iPSCs Derived Cells
[75]	Rabies virus transport, electrophysiology, calcium transients, microarray	Reduced neuronal connectivity, maintained synaptic function, altered gene expression in glutamate, cAMP, BMP, and WNT pathways; loxapine application normalizes alterations
[76]	Potassium-induced depolarization, RNA-seq	Reduced activity-dependent transcription, coexpression modules are enriched for GWAS risk variants
[77]	RNA-seq, reporter assay	Differentially expressed genes from WNT, SHH, BMP, and G-protein coupled signaling
[78]	Cell imaging, HPLC, mitochondrial assays	Impaired dopaminergic differentiation and glutamatergic maturation, mitochondria show reduced membrane potential, respiration, and connectivity, and uneven network structure
[79]	Potassium-induced depol-arization, HPLC, ICC	Increased basal and activity-dependent cortical secretion of dopamine, epinephrine, and norepinephrine
[80]	ICC	SCZ/control-iPSCs differentiate equally well into FOXA2-positive midbrain dopaminergic neurons
[81]	WCPC, calcium transients	Delayed differentiation of NPCs into DG-like neurons, reduced spontaneous neurotransmitter release
[82]	RNA-seq, SCS, WCPC, MEA	Reduced spontaneous spike and network bursts in mature, but not in early, SCZ iPSC-DG-CA3 circuits
[83]	Oxygen consumption, ROS production	Increased extra-mitochondrial oxygen consumption and ROS production that is normalized by valproate
[70]	Microarray, mass spectrometry	Abnormal gene and protein expression related to cytoskeletal remodeling and oxidative stress, impaired NPC migration, differentiation, and mitochondrial membrane potential, and increased ROS levels
[84]	Mitochondria transfer	Improved mitochondrial function and glutamatergic differentiation following mitochondria transfer
[85]	Expression studies in iNs, electrophysiology	miR-137 downregulates presynaptic plasticity genes and vesicle secretion in iNs
[55]	ATAC-seq, gene editing, SCS	Modification to a non-risk allele increases *MIR137* expression and reduces neuronal maturation
[86]	Nanostring, RNA-seq, mass spectrometry	Reduced miR-9 expression inhibits outgrowth migration of NPC neurospheres
[87]	RNA-Seq, small RNA-seq, ChIP-Seq	Increased expression of miRNA networks, impaired miRNA dependent gene regulation

Abbreviations are: ATAC-seq, sequencing assay for transposase-accessible chromatin; BMP, bone morphogenetic proteins; ChIP-Seq, chromatin immunoprecipitation sequencing; iN, induced neurons; HPLC, high pressure liquid chromatography; ICC, immunocytochemistry; MEA, multiple-electrode array; Nanostring, digital expression profiling; NPC, neuronal progenitor cell; RNA-seq, RNA sequencing; ROS, reactive oxygen species; SHH, sonic hedgehog; SCS, single cell sequencing; WCPC, whole cell patch clamp; WNT, Wingless.
